# Age-Standardized Mortality Rates in the Caribbean: One Source, Three Different Interpretations

**DOI:** 10.1200/JGO.18.00010

**Published:** 2018-08-02

**Authors:** Nicholas G. Wolf, Camille Morgan, John S. Flanigan

**Affiliations:** Center for Global Health, National Cancer Institute, Rockville, MD

## Abstract

**Purpose:**

A recent publication in *Morbidity and Mortality Weekly Report* (*MMWR*) provided the opportunity to calculate differences in published cancer mortality estimates for Caribbean jurisdictions from three organizations: *MMWR*, the International Agency for Research on Cancer, and the Institute for Health Metrics and Evaluation. This comparison study serves to educate end users of these data.

**Methods:**

We downloaded the publicly available cancer mortality estimates for 15 jurisdictions and the United States from the three organizations. We compared reported age-standardized mortality rates for each jurisdiction and calculated the range among estimates for each jurisdiction. We repeated this analysis after applying the same world population standard to all estimates.

**Results:**

For males, the ranges of the Caribbean estimates were between 49% (Grenada and Trinidad) and 201% (US Virgin Islands) of the *MMWR* value, with an average of 88%. For females, the ranges were between 15% (Trinidad) and 171% (US Virgin Islands) of the *MMWR* value, with an average of 64%. After all estimates were compared using the same population standard, the ranges of the Caribbean estimates for males were between 6% (Grenada) and 111% (US Virgin Islands) of the *MMWR* value, with an average of 34%. For females, the ranges were between 7% (Grenada) and 97% (US Virgin Islands), with an average of 28%.

**Conclusion:**

The use of different standard populations complicates comparisons across organizations. Data modeling does not completely compensate for quality of source data, as our analysis demonstrated by the differences in mortality rates despite the good quality of the vital registration in the Caribbean.

## INTRODUCTION

The growing burden of cancer in low- and middle-income countries (LMICs) has gained attention in recent years. To address this burden, health policymakers rely on functioning health information systems to generate the critical data needed to guide health policy decisions.^1^ Reliable and timely data are essential to produce efficient health policies, establish research priorities, and monitor the effectiveness of cancer control strategies.^2-5^ Mortality data, as an indicator of the most serious outcome of cancer, are a useful metric for both describing burden and evaluating efforts in prevention, early diagnosis, and treatment.^2^ The civil registration and vital statistics system (CRVS) of a country is the administrative recording of the occurrence and characteristics of vital events such as death and is considered to be the optimal source of mortality data.^6,7^ A CRVS aims to provide medically certified, cause-specific mortality information on a continuous basis and is intended to cover the entire population. Despite its importance, the WHO estimates that nearly half of LMICs do not have adequate CRVSs^7^ and that nearly two thirds of the deaths in the world go unregistered.^3,7^ Inadequate legislation, the logistic difficulties of collating information from different governmental databases, and the challenges of obtaining medically accurate causes of death from those who complete death certificates all contribute to this reality.^4^ The lack of improvement in strengthening CRVSs has been characterized as “the single most critical development failure in the last 30 years.”^8^^(p1526)^

The *Morbidity and Mortality Weekly Report* (*MMWR*) publication is prepared by the Centers for Disease Control and Prevention and “is the agency’s primary vehicle for scientific publication of timely, reliable, authoritative, accurate, objective, and useful public health information and recommendations,”^9^^(p1)^ including cancer mortality data from CRVSs. In addition to *MMWR*, organizations such as the International Agency for Research on Cancer (IARC) and the Institute for Health Metrics and Evaluation (IHME) publish estimates on the cancer mortality rates for countries around the world based on CRVS data reported to the WHO. To overcome the gaps in data quality and coverage, IHME and IARC have developed and refined modeling methodologies to improve the accuracy of mortality estimation. Their respective publications and interactive Web sites are valuable resources for policymakers, scientists, and other end users of cancer mortality estimates, from guiding national cancer control efforts to understanding the disease burden in context.

However, the uncertainty and variance inherent in modeling resulting from the different assumptions and methods used mean that the estimates generated do not always align.^10^ The three organizations in this analysis, *MMWR*, IARC, and IHME, have similar missions: *MMWR* publishes public health recommendations, IARC states that its estimates “aim to provide the evidence and impetus for developing resource-contingent strategies to reduce the cancer burden worldwide,”^11^^(pE359)^ and IHME regards timely mortality data as an “important impetus for public policy action.”^12^^(p2071)^ A comparative analysis of the data from these three organizations is important to inform end users who would use them as a basis for health policy reform.

In December 2016, Razzaghi et al^13^ published an analysis in *MMWR* of cancer mortality in Caribbean islands using data taken directly from the Caribbean Public Health Agency (CARPHA) and without modeling adjustments. The publication presents an opportunity to understand the differences in cancer death estimation among the two modeled sources of IHME and IARC and the empiric data source of *MMWR*. Caribbean mortality data are considered to be of good quality when compared with other LMIC regions.^3,7,14^ Therefore, large differences in Caribbean estimates resulting from modeling adjustments would suggest caution when interpreting estimates for LMIC regions of even lower data quality. In this study, we compare the age-standardized mortality rates (ASMRs) for 15 Caribbean islands and the United States published by the IHME Global Burden of Disease 2015 study,^15^ the IARC GLOBOCAN project,^11^ and the Razzaghi et al *MMWR* publication. This analysis illustrates the sources of variation among the methodologies for estimating disease burden to guide end users in their application of these data.

## METHODS

### Selection of Cancer Sites and Jurisdictions for Comparison

The *MMWR* report published ASMRs for all cancers and the top 10 cancers by cause of death for 23 Caribbean jurisdictions and the United States. The ASMRs for all cancers were compared with the corresponding estimates provided by IARC GLOBOCAN and IHME Global Burden of Disease for the jurisdictions available in each model. We focused on all cancer estimates in this study because the values are the largest and are not subject to disease-specific variability. Most recent IARC estimates are for the year 2012, so IHME estimates for 2012 were selected for the most appropriate comparison. Saint Vincent and the Grenadines was not analyzed for male cancers; these data were excluded from the *MMWR* report because of small numbers.

### Data Retrieval

IARC data were retrieved from the IARC Global Cancer Observatory site.^16^ The age-standardized rates of the selected cancers were downloaded for each available Caribbean country for each sex. IHME data were retrieved from the IHME Global Health Data Exchange site.^17^ For the year, 2012 was selected, and the age-standardized rates of the selected cancers were downloaded for each available Caribbean country for each sex. IHME is the only organization to publish CIs with its estimates, and these were included in the figures. Age-specific cancer mortality rates were also downloaded from IHME by selecting all 5-year intervals from 1 to 4 to 80+.

### Comparison of Estimates

**Fig 1:**
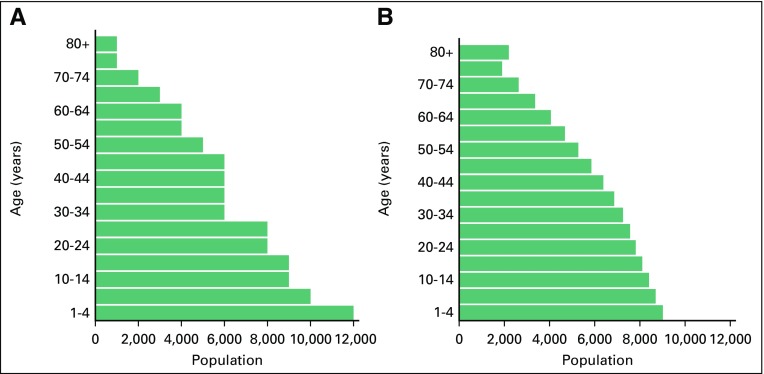
Distribution of (A) Segi^19^ and (B) Institute for Health Metrics and Evaluation standard populations (populations of 100,000). Data adapted.^18,19^

The three organizations use different world standard populations for the calculation of their ASMRs, and this difference is accounted for in our comparison. IHME uses a world standard population of its own derivation, which is a variation of the 2001 WHO world standard population with updated numbers to more accurately reflect the current global age distribution.^18^ IARC and *MMWR* use the Segi^19^ world standard population, which was introduced in 1960 and is significantly different from that of IHME (Fig 1).

We present the published ASMRs from each of the three organizations as an end user would access them, despite the differences in the world standard population used. We applied the Segi^19^ population weights to crude ASMRs downloaded from IHME and compared the ASMRs again, with all estimates using the same standard population. ASMRs are calculated by taking age-specific population weights, multiplying them by the corresponding age-specific cancer mortality rates, and then summing them to produce the ASMRs. Descriptions of the history, derivation, and calculations of ASMRs can be found in the report by Ahmad et al.^20^ Countries in the ASMR figures are ordered by descending magnitude of the *MMWR* estimate, with estimates for the United States shown last.

### Metric for Comparison

The range of the estimates was calculated by taking the difference between the maximum and the minimum values from the three organizations for each country. The range was then divided by the *MMWR* value to produce a measure of the maximum amount the estimates differed as a proportion of the empiric value *MMWR* represents: (maximum estimate − minimum estimate)/MMWR value.

The *MMWR* estimates were used as a baseline because they are not adjusted by modeling. This calculated measure reflects the uncertainty around the estimate and how much the models alter the empiric estimate in their attempt to capture the true mortality rate of the population.

## RESULTS

Table 1 summarizes the countries available from each data source, the years used to generate the estimates, and the quality of the mortality data. Of the 24 jurisdictions in the *MMWR* report, 16 are available in the IHME database and nine in IARC. ASMR estimates from the *MMWR* report reflect a variable 5-year interval listed in Table 1.

**Table 1:**
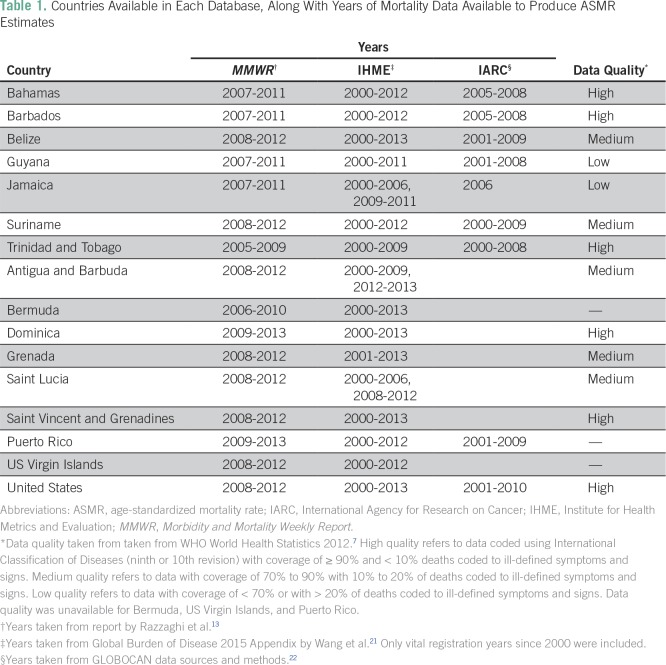
Countries Available in Each Database, Along With Years of Mortality Data Available to Produce ASMR Estimates

**Fig 2:**
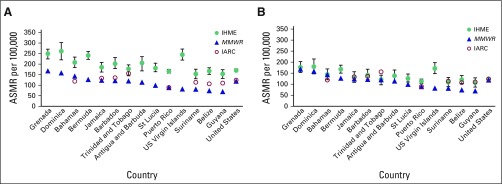
(A) Published estimates of age-standardized mortality rates (ASMRs) and (B) estimates standardized to Segi^19^ world population for all cancer sites for males from Institute for Health Metrics and Evaluation (IHME), *Morbidity and Mortality Weekly Report* (*MMWR*), and International Agency for Research on Cancer (IARC).

Figures 2A (males) and 3A (females) highlight the differences among estimates published by IHME, IARC, and *MMWR*. In all jurisdictions, for both men and women, the IHME estimate was the highest of the three ASMR estimates.

**Fig 3:**
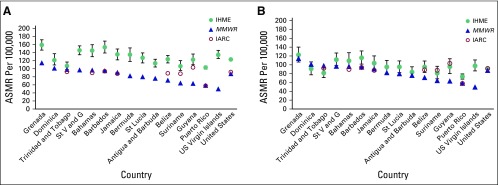
(A) Published estimates of age-standardized mortality rates (ASMRs) and (B) estimates standardized to Segi^19^ world population for all cancer sites for females from Institute for Health Metrics and Evaluation (IHME), *Morbidity and Mortality Weekly Report* (*MMWR*), and International Agency for Research on Cancer (IARC). NOTE. Scale of *y*-axis is different from that in Figure 2. St V and G, Saint Vincent and the Grenadines.

For males, the ranges of the Caribbean estimates were between 49% (Grenada and Trinidad; range, 82 and 58 deaths per 100,000, respectively) of the *MMWR* value and 201% of the *MMWR* value (US Virgin Islands; range, 163 deaths per 100,000). The average of the Caribbean ranges as proportions of the *MMWR* value was 88%. The range of the US estimates was 44% of the *MMWR* value (51.6 deaths per 100,000). For females, the ranges of the Caribbean estimates were between 15% (Trinidad; range, 14.7 deaths per 100,000) of the *MMWR* value and 171% of the *MMWR* value (US Virgin Islands; range, 84.9 deaths per 100,000). The average of the Caribbean ranges as proportions of the *MMWR* value was 64%. The range of the US estimates was 41% of the *MMWR* value (35.8 deaths per 100,000).

To correct for the methodologic difference between the three organizations related to the choice of world standard population, we compared all ASMRs using the Segi^19^ world standard population (Figs 2B and 3B). Using the same standard population clearly has an impact on the numbers produced, because the average ranges decreased by 64% and 59% for males and females, respectively. IHME is no longer consistently the highest estimate (Table 2).

**Table 2:**
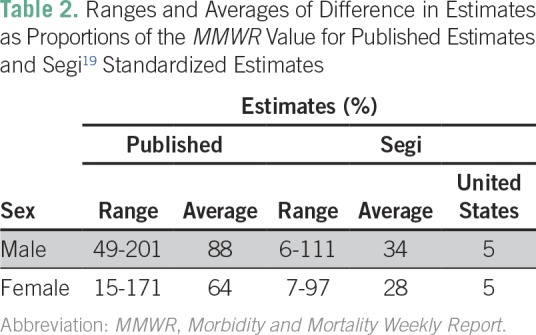
Ranges and Averages of Difference in Estimates as Proportions of the *MMWR* Value for Published Estimates and Segi^19^ Standardized Estimates

For males, the ranges of the Caribbean estimates were between 6% (Grenada; range, 9.5 deaths per 100,000) of the *MMWR* value and 111% of the *MMWR* value (US Virgin Islands; range, 89.9 deaths per 100,000). The average of the Caribbean ranges as proportions of the *MMWR* value was 34%, and the range of the US estimates was 5% of the *MMWR* value (5.5 deaths per 100,000). For females, the ranges of the Caribbean estimates were between 7% (Grenada; range, 14.7 deaths per 100,000) of the *MMWR* value and 97% of the *MMWR* value (US Virgin Islands; range, 48.1 deaths per 100,000). The average of the Caribbean ranges as proportions of the *MMWR* value was 28%, and the range of the US estimates was 5% of the *MMWR* value (4.5 deaths per 100,000).

The potential causes of variation across the three organizations are listed in Table 3, including data sources, modeling techniques, imputation and redistribution of ill-defined codes, and region-specific nuances, such as small populations.

**Table 3:**
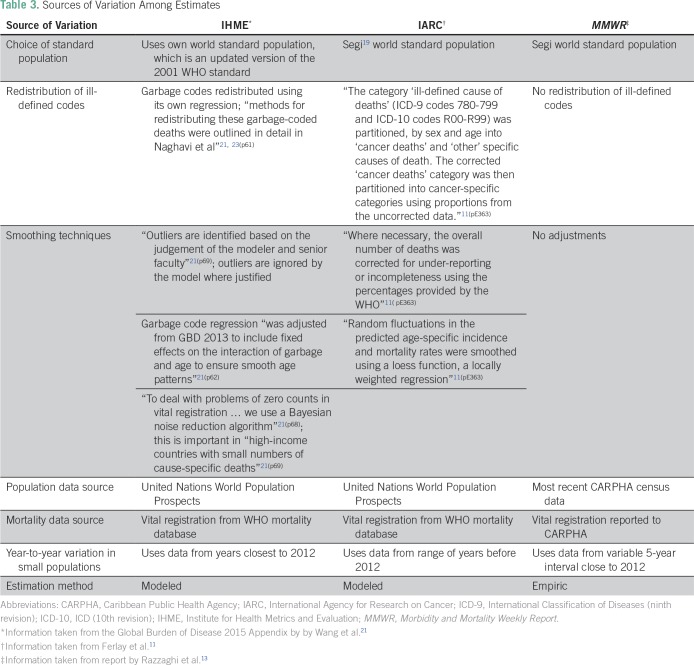
Sources of Variation Among Estimates

## DISCUSSION

Figures 2A and 3A present a striking image of the differences in the published cancer ASMR estimates available to end users. Without an understanding of the differences in standard population, IHME estimates seem more than double the *MMWR* estimates in several jurisdictions. The ranges of the ASMRs were on average 88% and 64% of the *MMWR* value for males and females, respectively, because of differences in the assumptions and methods of the three organizations. Much of this discrepancy can be explained by the choice of standard population, which, when taken into account, drops the average range of the estimates as proportions of the *MMWR* value from 88% to 34% for males and from 64% to 28% for females. ASMRs are used to compare mortality rates between populations that may have different age distributions, but there is no agreed upon international standard population to use for this calculation. IHME has developed its own standard population distribution to reflect the changing age-specific distribution of the global population more closely.^21^ The value of using this updated population distribution is that it prevents age-specific events from being weighted disproportionately.^20^ As the structure of the global population shifts over time, an outdated standard population no longer reflects the realities of the average country.

However, use of different standard populations produces different absolute values for ASMRs, and therefore, estimates from different standards cannot be compared directly.^20,24^ Updating the standard population to resemble the global population structure more closely has disadvantages. Because comparisons of mortality rates are useful across populations, time, and data sources, changing the standard population limits historical and lateral comparisons. Additionally, Bray et al^24^ demonstrate that Segi^19^-standardized ASMRs perform as well as the updated standard population rates as comparative measures of relative risk. Therefore, changing the standard population adds no utility to ASMRs as comparative measures, and the existence of multiple standards risks misinterpretation by end users of the data. It would be advantageous to establish a consistent standard population for end users, because users are cautioned not to mix ASMRs from data sources using different standard populations.

A useful compromise would be to present the crude mortality rate alongside an age-standardized rate calculated from a static population standard. The crude rate would reflect country-specific cancer burden and serve to contextualize the ASMR. However, presenting both values would double the number of data and would rely on the end user’s ability to make use of both data together.

Even after correcting for different standard populations (Figs 2B and 3B), differences still exist among the estimates, possible sources of which are summarized in Table 3. The ranges of estimates for the Caribbean jurisdictions were on average 34% and 28% of the *MMWR* value for males and females, respectively. One contributing factor to the observed differences is the quality of the death codes of the vital registration system of each country. Some portion of these codes are either missing or invalid, or the codes are considered garbage codes, a term describing instances in which the cause of death assigned could not or should not be used as the underlying cause of death. The *MMWR* article reports that for Caribbean countries, between 2.3% and 12.9% of the cause of death codes were invalid, missing, or unknown (median, 12% for countries reporting to WHO in 2005^14^), and the three organizations handle these data in different ways. The *MMWR* estimates make no corrections for these missing or garbage codes. IARC and IHME each have their own algorithms both to correct for missing data and to redistribute garbage codes to likely causes of death.^11,21^ These algorithms also serve to rein in outliers and smooth over fluctuations in predicted age-specific mortality (Table 3). The deviation between the modeled estimates highlights the importance of having mortality data from high-quality sources. Strengthening the vital registration system, including training for the correct completion of death certificates, as well as strengthening pathology and cancer diagnostics, reduces the frequency of missing and garbage codes. In turn, this limits uncertainty and error when redistributing these codes. Additionally, cancer registries are needed to properly capture local disease variation that may be smoothed over by these algorithmic techniques. Cancer registries capture heterogeneous characteristics of cancer tumors and allow for comparisons of different populations at the subnational level.

Other possible sources of variation include year sampling and the source of the mortality and population data. Table 1 lists the different years used by IHME, IARC, and *MMWR* to produce their respective ASMRs. Cancer death rates generally do not change rapidly over time,^21^ but cancer mortality from small populations such as those found in the Caribbean islands is subject to some amount of year-to-year variation. For mortality and population data, IARC and IHME use the WHO mortality database and the United Nations World Population Prospects. *MMWR* uses the civil registration data and population census data reported directly to CARPHA from the Caribbean countries. CARPHA sends its cancer mortality data to the Pan American Health Organization, which subsequently shares it with the WHO to be entered into the WHO mortality database, but the data could be subject to different methods of cleaning or correction for ill-defined codes during this transition. Ultimately, all three sources get their mortality data from a country’s civil registration, but they access it at different points along the data-sharing chain.

Given the differences in methodology outlined here, which data source should end users use for their cancer control needs? Although it is difficult to prove that one source is universally superior, the answer depends on the nature of the question asked. For example, data from *MMWR* are unmodeled, so the numbers most accurately reflect cancer deaths captured by a country’s health system. These data could be used to track infrastructural improvements in the vital registration system. Because of their exhaustive statistical methodology, data from IHME are more appropriate than those from *MMWR* for comparisons of countries with low data quality. The numbers from IHME in the Caribbean region tend to be higher than those from the other sources for a given country, which could be useful when advocating for the cancer burden. IARC specializes in cancer data from 2012, so its data are most appropriate for cancer mortality comparisons across countries for that time period.

Health data inform health policy, and large uncertainty ranges around an estimated value present a challenge to policymakers. The Caribbean is considered to have good-quality vital statistics when compared with other LMIC regions, so it is reasonable to expect even more variation for regions with lower-quality source data where more modeling adjustments are made. Regions with limited-quality source data are also more likely to rely on modeled estimates from independent organizations for their health policies. In September 2011, world leaders committed to the formation of the Noncommunicable Disease Global Monitoring Framework with the goal of reducing premature mortality from all noncommunicable diseases by 25% by 2025.^25^ The WHO estimates that only one in five countries can presently report such data with high levels of completeness and coverage,^26^ making tracking progress toward this goal difficult and highlighting the need to strengthen CRVSs around the world. Initiatives such as Bloomberg’s Data for Health^27^ and the Global Civil Registration and Vital Statistics Scaling Up Investment Plan^28^ have been launched to improve the global quality of vital statistics, but more efforts are needed.

The use of different standard populations complicates comparisons of ASMRs among organizations and across time. An end user of epidemiologic data produced by IHME, IARC, and *MMWR* should understand the differences in methodologies when using these data. Data modeling does not completely compensate for quality of source data, as our analysis demonstrated by the differences in mortality rates despite the good quality of vital registration in the Caribbean. The magnitude of the differences suggests caution for policymakers who would use these data to inform health policies. Source data quality is an important component of accurate estimation, and cancer mortality source data can continue to be improved by strengthening data collection, vital registration, and pathology.
